# Future gaps in the public provision of health services in Austria? A mixed-methods analysis for specialists in internal medicine

**DOI:** 10.1186/s12960-025-01013-3

**Published:** 2025-08-13

**Authors:** Clemens Zech, Monika Riedel

**Affiliations:** Institute for Advanced Studies, Vienna, Austria

**Keywords:** Workforce projection, Internal medicine, Stock-flow models, Gap analysis, Double aging

## Abstract

**Background:**

Population growth and aging are likely to increase demand for healthcare providers, even in countries with high provider-to-population ratios. To plan appropriate policy measures, detailed information on supply and demand trends in the physician workforce for each medical specialty is essential. This article assesses the current supply of all specialists in internal medicine (general and subspecialties) in Austria and projects future supply and demand until 2035.

**Methods:**

Our analysis follows a framework for workforce planning consisting of four stages: (1) horizon scanning, (2) scenario generation, (3) workforce modelling, and (4) policy analysis. We use stakeholder workshops, interviews and an online survey (*n* = 484) in the first two stages. Future supply is modelled using stock-flow models, whereas demand is projected using regression modelling based on existing forecasts and extrapolations of historical care use. Different scenarios are used to account for uncertain developments. The two main settings of care in Austria—public hospitals and publicly financed outpatient care—are modelled separately.

**Results:**

Overall, no severe shortage of specialists in internal medicine is expected in Austria until 2035. However, our analysis suggests that the two settings of care will experience very different developments: while the gap between supply and demand in public hospitals is expected to be small (± 5 percent), the supply of specialists in publicly financed outpatient care is projected to fall between 10 and 25 percent short of demand.

**Conclusions:**

Without major reforms, capacity constraints will likely affect the publicly financed outpatient sector, hindering the desired shift from inpatient to outpatient care or driving patients into the private outpatient sector where higher user charges apply. Therefore, it is essential for policy makers to incentivize physicians to work in publicly financed outpatient care. Increasing enrolment in medical schools is not a suitable policy measure, as no significant shortage of specialists in internal medicine is expected overall.

**Supplementary Information:**

The online version contains supplementary material available at 10.1186/s12960-025-01013-3.

## Background

The simultaneous ageing of the health workforce and rising care needs due to population ageing put health systems across the globe under pressure. This also applies to wealthy countries with more health workers, medical equipment and other health-related resources per capita compared to lower-income countries. National characteristics can aggravate the current pressure, as is the case in Austria, which usually ranks in top positions regarding the availability of health resources per capita. However, in contrast to many other European countries, the number of graduates from medical schools in Austria is now lower than it was a decade ago [1] because enrolment in medical schools was restricted later than in many other countries. In Germany, for instance, a strict *Numerus Clausus* had already been installed in 1968, in France in 1971, in Switzerland in 1998, but in Austria only in 2006. Until then, enrolment in publicly financed medical schools in Austria had been open and free of tuition fees for everyone with a valid Austrian high school diploma or an equivalent diploma from a member state of the European Union. Only the increasing inflow of students from Germany led to the first entrance examinations and predefined numbers of first-year students in Austrian medical schools [2]. Furthermore, approximately one in three of these graduates leave Austria within the first 3 years, reducing the national availability of future physicians [3, 4]. However, the number of practising physicians per capita is still among the highest, and the number of graduates per capita is close to the average value in Europe [1].

This relative abundance of medical capacities might explain the absence of national-level planning of the health workforce related to future care needs, resulting in knowledge gaps in various dimensions: professional (all physicians vs. individual specialties), care setting (inpatient, hospital outpatient, private practice with or without contract with social health insurance (SHI)^1^), and geographical (Austria vs. its nine states). These shortcomings were also noted in an older ad hoc forecast [5], which had been triggered by regulatory changes for foreign students. Even the major planning instrument for the Austrian health system (*Österreichischer Strukturplan Gesundhei—ÖSG*) still abstains from quantifying overall staffing numbers for hospital care. Thus, for most health professionals, including physicians, little is known about whether future supply will suffice to meet demand in Austria and where coverage gaps might appear.

Despite the high number of physicians, patients are increasingly experiencing long wait times for outpatient appointments in publicly financed care and, to a lesser extent, in privately financed care. Additionally, it is becoming more difficult to find publicly financed practices that accept new patients [6]. These new developments are connected to a peculiar incentive structure: physicians often perceive SHI-contracted work in private practices as less attractive than work without SHI contracts and related regulations. Furthermore, a modernization of the SHI fee schedules is overdue [7]. Employment in a public hospital can be combined with uncontracted work in private practice, which has recently received additional interest due to the EU working time regulation [6, 8]. As a result, in several regions, SHIs and hospitals increasingly struggle to contract physicians for public health care, albeit to differing degrees depending on the field of specialization [9].

As of yet, problems in contracting physicians in the field of internal medicine (IM) have been the exception. In 2015, the national training programme for IM was split into 11 subdisciplines: general IM and ten specializations, such as “IM and cardiology”.^2^ With increasing specialization and low incentives to provide contracted outpatient services, it is uncertain whether the growing need for specialists in publicly financed IM can be met in the coming years. Long and costly education periods make planning for health workers especially relevant because sudden gaps cannot be filled in the short term [5].

On an international level, analyses applying more sophisticated approaches rather than mere provider-to-population ratios have been published but concentrate on a small group of countries (Canada, the UK and the US), whereas regular analyses with sufficient scientific rigour are often lacking for many—even rather high-income—countries. This is often due to the data requirements that such approaches necessitate [11]. An elaborate planning exercise for a country with a health system (and data infrastructure) that deviates from the aforementioned group of countries can therefore be seen as a valuable addition to the health care planning literature.

This article aims to provide insights into the likely development of supply and demand for specialists in IM in Austria until 2035. To address this topic, we define IM in a broad sense, i.e. encompassing all 11 subspecialties, as the various subdisciplines are not yet sufficiently reflected in the data on health workforce and utilization.

## Materials and methods

Our models use several demand- and supply-side projections to conduct a national gap analysis for specialists in IM in Austria for the years 2023–2035. In particular, we study the provision of care in two distinct settings: public hospitals in Model 1 (M1) and SHI-contracted outpatient care in Model 2 (M2). We project supply using stock-and-flow models, which are widely used in the literature to track workforce inflows and outflows using arithmetic calculations [12]. Demand is estimated using regression modelling based on ÖSG projections (M1) and linear extrapolation of historical utilization data (M2). The gap between supply and demand is calculated by holding case-to-provider ratios constant from the base year 2022 through 2035. Finally, simple population-to-provider approaches are used for demand projections to assess robustness.

Our dataset combines detailed, high-quality data from multiple Austrian institutions. Data on the demographics of specialists in IM as well as their work setting are provided by the Austrian Medical Chamber, whereas data on the historical utilization of outpatient care are provided by the Federation of Social Insurances, which cover all three major health insurers and thus more than 99% of the Austrian population [13]. In addition, we use projections for hospital utilization from the ÖSG and population forecasts from Statistics Austria [14]. For our analysis, we follow the Robust Workforce Planning Framework, established by the Centre for Workforce Intelligence in the UK between 2010 and 2016. In this framework, horizon scanning is used to identify trends and uncertain factors that may influence supply and demand. This information is then used to develop several plausible scenarios, and workforce supply and demand are modelled for each scenario to identify gaps in the provision of care. Finally, policy implications are discussed [15].

In the horizon scanning and scenario generation phase, we applied a mix of qualitative and quantitative methods. We started by reviewing previous literature and conducting descriptive analyses of our data [16]. Thereafter, stakeholder interviews and workshops with board members of the Austrian Association for IM (ÖGIM) were conducted to explore factors such as healthcare delivery trends, potential changes in training capacities and changing preferences of physicians. Finally, we conducted a survey among all ÖGIM members to derive a quantitative range for these developments. A total of 484 responses were collected, which represent 15 percent of the 3200 ÖGIM members contacted via email. Together with expertise from an earlier analysis of a subspecialty of IM in Austria [17], this information was used to develop plausible narratives for the future provision of care. In stakeholder workshops with ÖGIM board members and meetings with our research group, several potential future scenarios were defined. The resulting scenarios are described in the results section and form the basis for our analysis of workforce supply and demand.

**Fig. 1:**
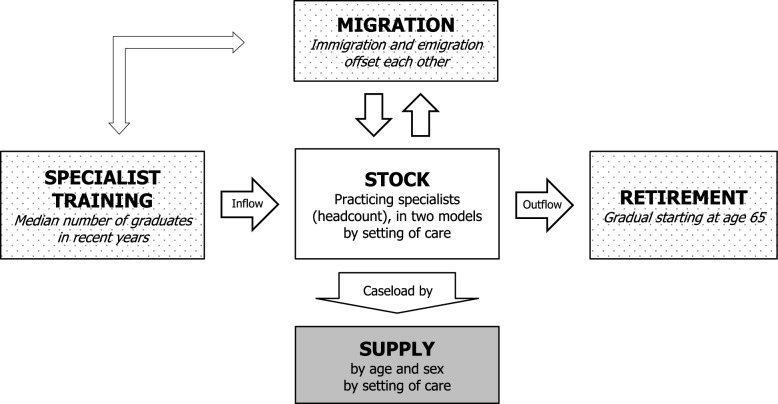
Stock-and-flow supply model overview. Source: own elaboration

We use a stock-and-flow model to forecast the supply of specialists for IM in various scenarios by estimating annual workforce inflows and outflows (see Fig. 1); the assumptions of our model are summarized in Table 1. We assume a constant inflow of 230 graduates per year on the basis of the median number of graduations from specialist training over the last 7 years, as there were no significant changes in training capacity. We distribute graduates proportionally between public hospitals (M1) and outpatient care settings (M2) on the basis of the overall distribution in the base year 2022. To account for a possible continuation of the trend toward decreasing interest in SHI-contracted work, we also include a scenario with reduced inflow into this setting in M2. Retirement is modelled as a gradual process over 3 years, starting from age 65. Specifically, in each cohort, 50% retire at age 65, half of the remaining cohort (a quarter of the original cohort) at age 66, half of the remaining cohort at age 67, and all remaining specialists retire at age 68. While no detailed data for the retirement ages of physicians in Austria are available, an international systematic review shows that physicians commonly retire between 60 and 69 years of age [18]. Even though the retirement process is usually spread over a period longer than three years, data from the Austrian SHI show that in the publicly financed outpatient sector, physicians past retirement age tackle considerably smaller caseloads than younger colleagues do, and retirement patterns therefore do not significantly change the overall amount of care delivery. Finally, we assume that inflows and outflows from immigration and emigration offset each other, which is in accordance with earlier work [5] and data provided by the registration authority (ÖÄK). Unfortunately, our model cannot consider individuals exiting the workforce before retirement due to a lack of data. The use of aggregated data in stock-and-flow models means that individual preferences and heterogeneous behaviour cannot be captured—for example, when physicians respond to policy changes in diverse ways [12]. However, given the relatively short projection period of 12 years, we assume that these limitations are unlikely to significantly affect our results.

**Table 1:** Model parameters and assumptions

Common to both models					
	M1-hospital	M2-SHI	Basis	Data source	Projection
Inflow	230 graduates from specialist training per year		Median number of IM graduates 2015–2022	ÖÄK	Constant
Outflow	Retirement: 50% retire at 65; 25% of the original cohort at 66; 12,5% at 67; and all remaining retire at 68		Specialist activity by age in 2022	ÖÄK	Constant
Migration	Immigration = emigration		Registration data	ÖÄK	Constant

Specific to each model					
	M1-hospital	M2-SHI	Basis	Data source	Projection
Inflow	66% of graduates per year (*n* = 152)	13% of graduates per year (n = 30)	Share of physicians in the respective setting in 2022a	ÖÄK	Constant
Utilization (proxy for demand)	Number of hospital stays for selected case groups in 2019, 2021 and 2030	Number of cases treated by specialists for IM between 2012 and 2019		M1: ÖSG (GÖG); M2: Federation of Austrian Social Insurance	Linear trend

^a^The remaining share of 21% of graduates per year (*n* = 48) works in other settings, most notably in noncontracted private practices

Our demand-side analysis follows a utilization-based approach. To project future healthcare utilization in the public hospital sector, we identify case groups relevant for IM in collaboration with ÖGIM representatives. For these groups, we sum the case numbers from the ÖSG for the years 2019 and 2021 and expected case numbers for 2030. Based on these values, we applied linear extrapolation to project demand until 2035. For the SHI-contracted outpatient sector, we conduct a linear extrapolation of historical case numbers between 2012 and 2019, omitting data between 2020 and 2022 due to COVID-19-related anomalies. Noncontracted physicians were excluded from our analysis because of a lack of robust data. However, according to information provided by ÖGK, approximately 87 percent of care is delivered within the SHI contract system, which suggests that the exclusion of noncontracted physicians does not significantly affect the overall findings. Finally, simple provider-to-population models are used in both models as robustness checks, which are based on the provider-to-population ratio in 2022 and total population forecasts from Statistics Austria.

## Results

The horizon scanning phase demonstrated that the most relevant trends affecting the future supply of specialists are shifts in physicians’ preferences for (1) working less than full-time and (2) choosing their setting of work. As a result of the scenario generation phase, which included the survey among ÖGIM members (see supplement Table 1), the following scenarios were defined. In the public hospital setting (M1), new specialists either increase their workload by 10% compared with current physicians (optimistic scenario), maintain the same workload (neutral scenario), or decrease their workload by 10% (pessimistic scenario). In the SHI-financed outpatient sector (M2), the same workload variations apply, and in addition, the availability of SHI-contracted specialists varies. In the neutral scenario, the number of SHI contracts remains stable, and positions are filled, whereas in the pessimistic scenario, either SHI contracts decrease, or positions go unfilled, reducing the inflow of specialists into this setting by 15% compared with the baseline year. As survey results suggest that an increase in the number of SHI contracts is unlikely, such a scenario was not modelled. For demand projections, the analysis uses one main projection in each model. Additionally, simple provider-to-population projections that do not consider expected changes due to ageing or healthcare delivery are used to assess robustness.

**Fig. 2:**
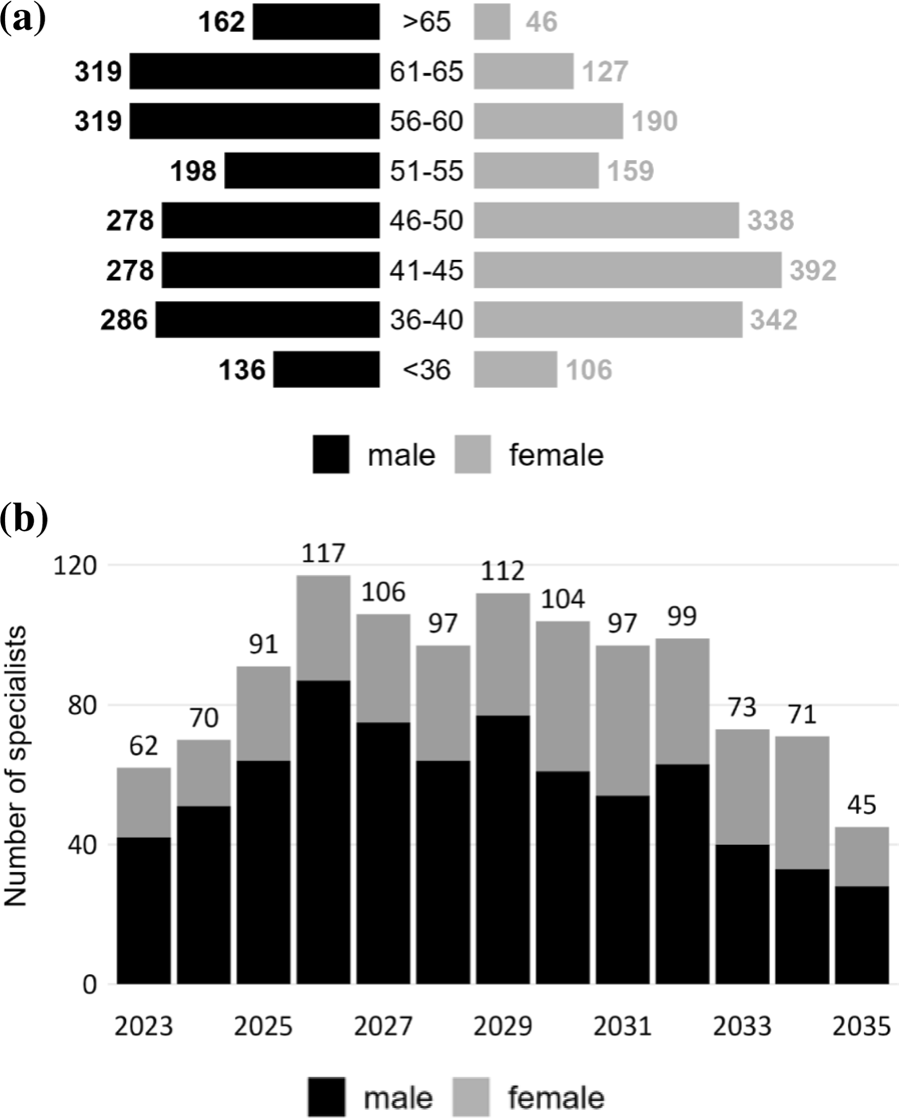
Specialists in M1 (hospital setting). (**a**) Age and gender distribution (2023); (**b**) number of specialists reaching age 65 per year

Figure 2a shows the age and gender distribution of specialists in M1, whereas Fig. 2b shows the annual number of specialists reaching age 65. A high number of retirements is expected between 2026 and 2032; however, the inflow of new graduates in M1 (152 specialists per year) is projected to exceed the outflow over the entire period. On the basis of ÖSG data, demand is projected to increase by approximately 18.5% until 2035. Assuming the workload per specialist does not deviate more than 10 percent from the base year, the gap between supply and demand is likely to remain within a range of ± 5 percent of demand (see Table 2).

**Table 2:** Projected gap between supply and demand of specialists in 2035 (absolute number of specialists shown in parentheses)^a^

MODEL 1: Public hospital setting		
Caseload per new specialist^b^, compared to base year	Gap (demand based on ÖSG data)	Gap (demand based on provider-to-population approach)
90% (pessimistic)	− 3.3% (− 137)	+ 10.2% (+ 373)
100% (neutral)	+ 1.0% (+ 43)	+ 15.1% (+ 553)
110% (optimistic)	+ 5.3% (+ 223)	+ 20.0% (+ 773)

MODEL 2: SHI-contracted setting				
Caseload per new specialist, compared to base year	Gap (demand based on SHI data)		Gap (demand based on provider-to-population approach)	
	(1) i^c^ = 0.85	(2) i = 1	(1) i = 0.85	(2) i = 1
90% (pessimistic)	− 26.8% (− 206)	− 20.6% (− 158)	− 20.2% (− 142)	− 13.3% (− 94)
100% (neutral)	− 22.2% (− 170)	− 15.9% (− 122)	− 15.1% (− 106)	− 8.2% (− 58)
110% (optimistic)	− 17.5% (− 134)	− 11.2% (− 86)	− 10.0% (− 70)	− 3.1% (− 22)

^a^Negative value denotes supply shortages, compared with usage/ratio in base year

^b^Scenario modelling concentrates on changes in new graduates from specialist training. A preference for reduced working time among new specialists by 10 percent then corresponds to a 10 percent reduction in the number of cases or patients treated and thus reduced productivity compared with the base year 2022

^c^i denotes the inflow of new graduates, share compared with the base year 2022

**Fig. 3:**
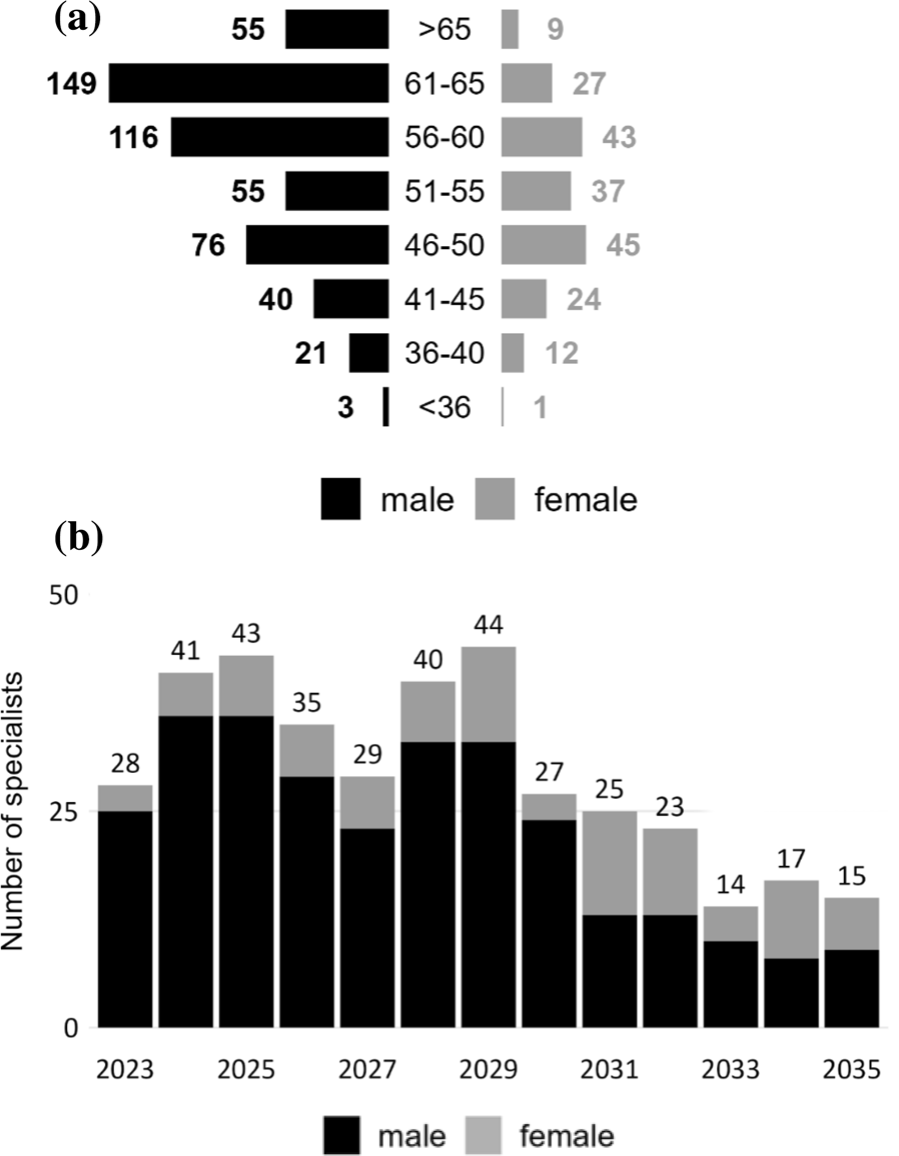
Specialists in M2 (SHI). (**a**) Age and gender distribution (2023); (**b**) number of specialists reaching age 65 per year

Similarly, Fig. 3a, b shows the data for specialists in M2. Compared with the public hospital setting, a larger share of contracted IM specialists is expected to retire over the next few years. By 2029, the average number of specialists turning 65 exceeds the number of IM specialists entering an SHI contract (30 specialists per year). Demand is projected to increase at the same rate as before the pandemic; on average, that is, by 13.6% per year. Depending on the assumptions, the gap in 2035 ranges between 11 and 27 percent of demand (see Table 2).

Finally, validation, sensitivity, and robustness checks are performed. The general structure of the model was validated in discussions with the ÖGIM board. As explained, we conduct deterministic sensitivity analyses by calculating outcomes using different parameter values, considering both optimistic and pessimistic scenarios. We find the model to be sensitive to factors such as variations in workload, even though these changes are applied to new entrants only. Differing retirement rules have little impact and are reported elsewhere [16]. As a robustness check, we project demand using simple provider-to-population approaches, which lead to significantly lower increases in demand in both models, as was expected due to population ageing. Therefore, these estimates serve as lower limits for demand in very optimistic scenarios.

## Discussion

The analysis suggests that until 2035, the gap between the supply and demand of specialists for IM in Austrian hospitals will be similar to the gap in the base year 2022; however, the gap will increase considerably with regard to SHI-contracted specialists. In the SHI setting, the supply of specialists in IM is modelled to fall up to 27 percent short of demand. The aggregated view, however, cannot show differences between subspecialties and regions, and larger gaps are likely to arise in some areas.

The larger gaps emerging in the SHI setting are in accordance with recent agent-based simulation forecasts [19]. While stock-and-flow models estimate annual workforce inflows and outflows, agent-based simulation models simulate the behaviour of individual workers, referred to as agents [12]. The workforce evolves over time as agents—each with unique characteristics—follow defined rules (e.g., retiring at a certain age). This allows for the modelling of more complex interactions. While we make different assumptions regarding the sectors physicians choose to work in based on their distribution across sectors in 2022, Popper and Rippinger [19] simulate physicians who make and reevaluate this choice over time, depending on their age and specialty. In addition, we used headcounts due to a lack of data on working times, whereas they estimated full-time equivalents based on expert opinions.

Current political and societal developments will likely result in rising workloads for SHI-contracted physicians, including specialists for IM: (1) health policy in Austria explicitly aims at shifting former inpatient care to less costly settings, i.e. outpatient clinics and, where possible, practices with SHI contracts, and (2) population ageing increases the complexity of care. Accordingly, a majority of our survey respondents expect the workload per IM specialist to increase by more than 10 percent during the forecast period, which would presumably further reduce the attractiveness of SHI contracts. This combination of parameters is roughly represented by the scenario resulting in a gap of 17.5 percent of demand (inflow 0.85 compared with the base year, cases per specialist 10 percent higher than in the base year). If, however, new specialists treat fewer patients than their experienced colleagues do, the gap would be even larger. As many physicians have already opted out of the public health system and run private practices without SHI contracts, a modernization of SHI fees could be used to counteract this trend. This could be integrated into a harmonization of the fee structure, which has not been implemented since the 13 largest SHI organizations were merged into three SHI organizations in 2020. The modernized fee structure would need changes in the incentive structure (e.g., more bundled payments and capitation rather than fee-for-service) to avoid unnecessary contacts and services that currently incentivize physicians to increase activity. The reason for contracted physicians’ discomfort with the current system is their low payment per service rather than their overall income, as a recent study suggested: noncontracted outpatient physicians on average earn less per month than their contracted colleagues do, but more per patient, and treat fewer patients [20].

Currently, workforce planning in Austria considers only enrolment in medical schools and is largely detached from future care needs. The number of positions available to medical graduates for training in a subspecialty depends on individual hospital management and funding, and it is estimated that approximately 10–15% of all training positions across all specialties are vacant [19]. Considering the long training periods in medical professions, it is essential to implement long-term national planning of training posts by specialty, including staff capacities of trainers. A lack of planning will likely impede needs-based medical care, particularly in IM, as this field is highly specialized. Without needs-based planning, Austria risks an increasing imbalance in specialist supply, depending on the attractiveness of the subspecialties of IM, including relative earnings and reputation.

Limitations include that, like most comparable work, we cannot measure supply against a medically sound needs variable but use current utilization data instead [11]. Considering the anecdotal but rich evidence on wait times for appointments with contracted specialists for IM, our estimates are likely to underestimate true need. Even though a trend toward reduced working hours was identified in the horizon scanning phase, our analysis relies on headcounts rather than full-time equivalents because information on physicians’ working hours is missing. Different scenarios account for the possibility that shifts in physicians’ working hours will continue; however, developments such as medical progress, digitalization and increased use of AI may affect the productivity of physicians but were not modelled. While the demographics of both providers and patients may vary by subspecialty, we cannot model supply and demand in each subspecialty separately. Owing to the long duration of specialty training programmes, in combination with the slow uptake of such changes in administrative data, this is likely to remain an open issue in Austria for the next few years. Due to missing data on workforce exits, we were also unable to consider individuals leaving the workforce pre-retirement in our models.

Another limitation is that we cannot fully account for workforce plasticity, especially between IM in a broad sense and other specialties, such as general medicine. IM specialists holding double qualifications (IM and GP) and working in the publicly financed health system as GPs were removed from the supply side in our analysis. Earlier work for gastroenterology, however, showed that in publicly financed outpatient practice, many GPs and some surgeons sometimes perform procedures or tasks that fall within the spectrum of gastroenterology. The number of such GPs declined slightly during the observation period (2015–2019), and we assume that it will continue to decline because of lower interest in general medicine than in specialties among graduates from medical schools. We have to assume, however, that “specialty overlaps” persist to some degree [17]. Finally, gender-specific differences in case load are not modelled. The case load per SHI-contracted specialist has been evaluated elsewhere and shows that among contracted IM specialists in Austria, median earnings for women are approximately 25 percent lower than those for men, proportional to their caseloads [20]. Age and gender distributions (see Figs. 2a and 3a) show that the gender balance is more even in younger age cohorts; however, no information about the gender balance among recent entrants into SHI contracts is available.

Previous studies on health workforce planning often fail to report detailed information on methods and data sources used for the analyses. In many cases, data from various sources need to be combined, which may hamper consistency [12]. For example, an analysis of 32 OECD countries found that the necessary data for projecting future workforce trends in the health sector are only available in one-third of the countries, even for broad, aggregate-level analyses such as physicians, GPs and midwives [21]. In contrast to cross-country analyses, national analyses can often access broader and more granular data. A strength of the present article is that it shows how comprehensive administrative data from very different sources and stakeholders can be harmonized to populate a modular model, resulting in a rich mosaic of the current landscape of care in one large medical specialty (internal medicine) while following the recommendations of a recent reporting guideline [12] for health workforce planning.

## Conclusion

While hardly any national physician capacity planning is available for Austria, this paper shows that the experienced scarcity of IM is likely to stabilize rather than increase. Ceteris paribus, capacity constraints will concentrate on the publicly financed outpatient sector, thus increasing private payments (by turning to the costly private sector) and hampering the political aim of shifting care from inpatient to outpatient settings. Incentivizing work in contracted outpatient care seems to fit better with expected supply and demand development than increased enrolment in medical schools, owing to the long education process and limited capacity for training positions in specialized care. Countermeasures are needed to avoid a concentration of problems in the SHI sector: the overly large Austrian hospital sector repeatedly has been criticized as being too large and resource-consuming, resulting in health policy intending to shift care from hospitals to physician practices [6]. While in IM specialists can still be contracted quickly when older specialists retire, other specialties of medicine already experience longer vacancies and rely even more on suitable planning models and databases to close existing gaps in information and, ultimately, medical care.

## Supplementary Information

Additional file 1.

## Data Availability

Survey results can be found in the supplementary material. The datasets used and/or analysed during the current study are available from the corresponding author upon reasonable request.

## Abbreviations

IM: Internal medicine
ÖGIM: Austrian Association for Internal Medicine (Österreichische Gesellschaft für Innere Medizin)
ÖGK: Austrian health insurance fund covering approximately 80 percent of the population (Österreichische Gesundheitskasse)
ÖSG: Main Austrian planning instrument for health care capacities and quality indicators (Österreichischer Strukturplan Gesundheit)
SHI: Social health insurance

## Footnotes

1 While almost every person living in Austria is in some way covered by social health insurance, not all physicians licensed to practice participate as providers in the public health system. SHI offers only a limited number of outpatient contracts, while physicians working without SHI-contract in the outpatient sector have to be paid out of pocket. Public hospitals usually work with salaried physicians. Throughout this paper, the terms ‘publicly financed outpatient care’ and ‘SHI-contracted care’ refer to the same type of service provision, as do ‘privately financed outpatient care’ and ‘noncontracted care’.

2 The national training program for IM was split into the following disciplines: General IM, IM and Angiology, IM and Endocrinology and Diabetology, IM and Gastroenterology and Hepatology, IM and Haematology and Internal Oncology, IM and Infectiology, IM and Intensive Care Medicine, IM and Cardiology, IM and Nephrology, IM and Pulmonology, IM and Rheumatology; see *Ärztinnen-/Ärzte-Ausbildungsordnung 2015* [10].
